# Complete Genome Sequence of Streptomyces sp. Strain BSE7F, a Bali Mangrove Sediment Actinobacterium with Antimicrobial Activities

**DOI:** 10.1128/genomeA.00618-18

**Published:** 2018-06-28

**Authors:** Ira Handayani, Shanti Ratnakomala, Puspita Lisdiyanti, Fahrurrozi, Mohammad Alanjary, Wolfgang Wohlleben, Yvonne Mast

**Affiliations:** a Department of Microbiology/Biotechnology, Interfaculty Institute of Microbiology and Infection Medicine, Faculty of Science, University of Tübingen, Tübingen, Germany; b Research Center for Biotechnology, Indonesian Institute of Sciences (LIPI), Cibinong, Indonesia; c Applied Natural Products Genome Mining, Interfaculty Institute of Microbiology and Infection Medicine, Faculty of Science, University of Tübingen, Tübingen, Germany; d German Center for Infection Research (DZIF), Tübingen, Germany

## Abstract

The strain Streptomyces sp. BSE7F, a novel Streptomyces strain isolated from Indonesian mangrove sediment, displays antimicrobial activities against Gram-positive bacteria, Gram-negative bacteria, and yeast. Bioinformatic analysis of the genome sequence revealed the occurrence of 22 biosynthetic gene clusters disclosing the secondary metabolite capacity of strain BSE7F.

## Abstract (precis)

The strain Streptomyces sp. BSE7F, a novel Streptomyces strain isolated from Indonesian mangrove sediment, displays antimicrobial activities against Gram-positive bacteria, Gram-negative bacteria, and yeast.

## GENOME ANNOUNCEMENT

Actinomycetes have proven to be an excellent source of antibiotics (1, 2). In recent years, bioprospecting of rare habitats has turned out to be an efficient way to discover novel antibiotic producers (3). Indonesia is one of the most species-rich countries in the world (4). This biodiversity may also be reflected by microbial species diversity. Thus, Indonesian soils should serve as an excellent source of novel antibiotic producers. However, due to the overexploitation of terrestrial actinomycetes, the search for new antibiotics is more promising in unique environments. One such habitat is represented by the mangrove ecosystem (5). Many rare and novel actinobacterial species have been isolated from mangrove samples and have been shown to be potent producers of new bioactive secondary metabolites (6–10).

In this study, a novel Streptomyces strain, designated BSE7F, was isolated from a mangrove sediment sample from Bali, Indonesia. BSE7F exhibits antimicrobial activity against selected Gram-positive bacteria (Bacillus subtilis, Micrococcus luteus, and Staphylococcus carnosus), Gram-negative bacteria (Escherichia coli), and yeast (Saccharomyces cerevisiae). Here, we present the annotated genome sequence of strain BSE7F and report on its biosynthetic potential for antibiotic production.

The BSE7F genome was sequenced using PacBio RS II technology. The total genome size of BSE7F is 7,510,161 bp with five contigs and a G+C content of 72.3%. Using CheckM marker gene analysis (11), genome completeness was determined to be 98.3% with 0.4% contamination. Genome annotation with Prokka version 1.12b (12) identified 6,880 coding sequences (CDSs), 79 tRNAs, and 21 rRNAs on the BSE7F genome. Phylogenetic analysis of the whole-genome sequence using RAxML Web servers (13) revealed that BSE7F is closely related to Streptomyces sp. HNS054 (14). Average nucleotide identity (ANI) searches were performed using MASH (15) against RefSeq (16) genomes, which resulted in one match with an ANI over 95%. This was confirmed via JSpeciesWS (17), which showed a 97.1% ANI to Streptomyces sp. HNS054. An ANI higher than 95% indicates that BSE7F belongs to the same species as Streptomyces sp. HNS054, a strain originally isolated from a marine sponge (14).

In order to identify the antibiotic gene clusters (BGCs) that are responsible for the bioactivity of BSE7F, the genome was analyzed using antiSMASH version 4.0 (18), which predicted 22 BGCs. Seven of these matched known clusters for desferrioxamine B (19), venezuelin (20), albaflavenone (21), alkylresorcinol (22), naphthyridinomycin (23), and ectoine (twice) (24) with 100% similarity and three clusters encoding resistomycin (25), hopene (26), and spore pigment biosynthesis with >80% similarity. The remaining clusters were predicted to encode 3 polyketide synthase (PKS)/nonribosomal peptide synthetase (NRPS) hybrids, 2 terpenes, 2 bacteriocins, 1 NRPS, 1 phenazine, 1 lassopeptide, 1 siderophore, and 1 aminoglycoside/aminocyclitol-terpene hybrid. Overall, the discovery of several unknown putative BGCs in BSE7F is providing a strong basis for further experimental studies on the antibiotic production capacity of BSE7F, which may lead to the discovery of novel natural compounds. These preliminary studies suggest that Indonesia’s mangrove soil is a promising source of novel bioactive compound producers.

### Accession number(s).

This whole-genome shotgun project has been deposited at DDBJ/ENA/GenBank under the accession number QEQV00000000. The version described in this paper is the first version, QEQV01000000.
